# Better effect of intrapleural perfusion with hyperthermic chemotherapy by video‐assisted thoracoscopic surgery for malignant pleural effusion treatment compared to normothermic chemoperfusion of the pleural cavity

**DOI:** 10.1002/cam4.4450

**Published:** 2021-12-01

**Authors:** Yejun Cao, Qiying Zhang, Zhiyuan Huang, Zhengjun Chai, Jie Liu, Jinyi Wang, Zhengliang Sun, Tian Zhao, Guangxue Wang, Guohan Chen, Yang Han, Qinchuan Li, Xuan Hong

**Affiliations:** ^1^ Department of Thoracic Surgery Tongji University School of Medicine Shanghai East Hospital Affiliated to Tongji University Shanghai China; ^2^ Shanghai Tangqiao Community Healthcare Center Shanghai China; ^3^ Research Center for Translational Medicine Tongji University School of Medicine Shanghai East Hospital Affiliated to Tongji University Shanghai China; ^4^ Department of Pathology Tongji University School of Medicine Shanghai East Hospital Affiliated to Tongji University Shanghai China

**Keywords:** curative effect, intrapleural perfusion with hyperthermic chemotherapy (IPHC), malignant pleural effusion (MPE), normothermic chemoperfusion of the pleural cavity

## Abstract

**Objective:**

The aim of this study was to assess the efficacy and safety of intrapleural perfusion with hyperthermic chemotherapy (IPHC) in treating malignant pleural effusion (MPE) compared to normothermic chemoperfusion of the pleural cavity (NCPC), and to investigate the better treatment to control MPE.

**Methods:**

Malignant pleural effusion patients were enrolled in the study and treated with NCPC or IPHC under video‐assisted thoracoscopic surgery (VATS). The chest drainage duration, clinical characteristics, and recurrence time of pleural effusion of patients were collected for statistical analysis. The chi‐squared test and the Fisher's exact test were applied to compare the distribution differences in categorical variables. Progression‐free survival (PFS) was estimated by the Kaplan–Meier method and was compared by the log‐rank test. The survival analysis was performed using the Cox proportional hazards method.

**Results:**

A total of 37 MPE patients were enrolled in this study. Twenty‐seven patients received NCPC and 10 patients received IPHC under VATS. Significant differences were found in pathological types (*p* = 0.011), chest drainage duration (*p* = 0.005), and remission rate (*p* = 0.009) between two different treatment groups. The chest drainage duration of IPHC under VATS was shorter than the NCPC group (*t* = 2.969, *p* = 0.005). The remission rate of MPE in IPHC group was better than the NCPC one (OR = 0.031, 95% CI: 0.002–0.507, *p* = 0.015). The result of the Kaplan–Meier method showed that IPHC group could significantly prolong the PFS of patients with MPE compared to NCPC group (log‐rank *p* = 0.002). Univariate cox regression analysis showed that patients with MPE in the IPHC group presented significant longer PFS than the NCPC group (HR = 0.264, 95% CI: 0.098–0.713, *p* = 0.009). Multivariate cox regression analysis further verified this conclusion (HR = 0.268, 95% CI: 0.096–0.753, *p* = 0.012).

**Conclusion:**

Compared to the NCPC, the IPHC under VATS presents a better control effect on MPE, shorter tube placement time, and longer complete remission time. For this reason, we recommend IPHC under VATS as the first‐line treatment for patients with MPE those who can tolerate minimally invasive surgery.

## Graphical abstract

There have been some reports on the efficacy of intrapleural perfusion with hyperthermic chemotherapy (IPHC) in the treatment of malignant pleural effusion (MPE), but few studies put emphasis on IPHC under video‐assisted thoracoscopic surgery (VATS) and the efficacy of different treatment regimens. This study aims to explore the curative effect of IPHC under VATS and normothermic chemoperfusion of the pleural cavity (NCPC) in the treatment of MPE, and to provide clinical experience for MPE.

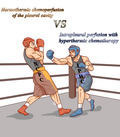

## 1 INTRODUCTION

Malignant pleural effusion (MPE) refers to the identification of tumor cells in pleural effusion or in pathological tissue taken from the patient with pleural effusion by pleural biopsy.^1^ The metastasis of advanced lung cancer and the pleural metastasis from any other primary tumors are the most important cause of MPE. In addition, the relatively rare malignant pleural mesothelioma is another important cause.^1^, ^2^ Common treatments for MPE include thoracic perfusion chemotherapy (TPC) and normothermic chemoperfusion of the pleural cavity (NCPC) with minocycline, human endostatin, and traditional Chinese medicine extracts such as cinobufosin and staphylococcal enterotoxin C injection.^2^, ^3^, ^4^, ^5^ However, there is no standard treatment for MPE patients currently, and the effectiveness of pleurodesis using these drugs has been criticized. Although platinum‐based NCPC has a certain control effect on MPE, pleural adhesion can be a side effect at the same time. Once large pleural effusion recurs, it will cause difficulties in the following treatment of MPE. Therefore, the medical science has searched for a treatment that can both effectively control MPE and improve the life quality of patients.

The treatment that uses hyperthermic chemotherapeutic drugs to perfuse the pleural cavity to reduce tumor cells, termed as intrapleural perfusion with hyperthermic chemotherapy (IPHC) has been applied in clinical.^1^, ^3^, ^6^, ^7^, ^8^ This therapy kills tumor cells by elevating the heat‐sensitive chemotherapeutic drug to the appropriate temperature (43–45°C) and recycling the drug in the pleural cavity by a closed circulation built in the extracorporeal circulation device. IPHC can raise and maintain treatment temperature effectively in the pleural cavity. Different temperature tolerance between normal tissues and tumor cells can not only achieve the therapeutic purpose by inducing apoptosis of tumor cells, but also protect normal tissues from the damage. IPHC makes an organic complementary effect between hyperthermia and chemotherapy infusion drugs, and increases the sensitivity of patients to chemotherapy. It can kill malignant tumor cells more effectively, improve the patient's quality of life, extend the patient's life, and at the same time reduce the side effects of chemotherapy. At present, some research had reported the efficiency of IPHC in controlling MPE. The curative effect is mainly achieved by heating the physiological fluid (mostly 0.9% NaCl solution) mixed with platinum drugs to 43–45°C, and continuous perfusion through the extracorporeal circulation machine to reduce malignant tumor cells in the thoracic cavity.^1^, ^7^ The study reported by Ba et al. showed that 100% of patients with MPE were controlled after accepting IPHC, and the average Karnofsky performance status (KPS) score was increased by 40%. At the same time, the survival time of MPE patients received IPHC was significantly prolonged, with a median prolonged time of 12.9 months.^6^ However, only a small number of MPE patients were used this therapy in clinical. The main reason is that most patients with MPE who cannot tolerate large‐scale surgery, are in the terminal stage of tumors, with poor nutrition and unstable vital signs. Therefore, less traumatic chest drainage combined with NCPC is used for symptomatic treatment for MPE. The IPHC under video‐assisted thoracoscopic surgery (VATS) used in this study combines the advantages of minimally invasive surgery, chemotherapy, and hyperthermia, which can achieve the purpose of diagnosis and treatment at the same time. Moreover, it is less traumatic, and most end‐stage patients with MPE can bear it.

Nowadays, reports on the efficacy of IPHC have been published, but few studies put emphasis on IPHC under VATS and the efficacy of different treatment regimens. This study aims to explore the curative effect between IPHC and NCPC, and to provide clinical experience for the treatment of MPE.

## 2 MATERIALS AND METHODS

### 2.1 Ethical approval

This study was conducted in accordance with the Helsinki Declaration of the World Medical Association (1964, revised in 2004), and the research program was approved by the Ethics Committee of Shanghai East Hospital Affiliated to Tongji University.

### 2.2 Study cohorts

Patients with MPE received IPHC or NCPC were enrolled in this study between January 2011 and May 2021 from Shanghai East hospital. The inclusion criteria are as follows: (1) Patients with MPE all confirmed by pleural cytology and/or biopsy; (2) Moderate to large MPE measured by B‐ultrasound or computed tomography (CT); (3) Age ≥20 years old, performance status (PS) 0–3, ALT <40 IU/L, AST <50 IU/L, serum creatinine <1.2 mg/dl, white blood cell >4 × 10^9^/L, and body temperature<37°C; and (4) The expected survival time is greater than 3 months. After obtaining informed consent, all MPE patients underwent NCPC after being intubated by ultrasound guidance or underwent IPHC under VATS.

### 2.3 Treatment

**FIGURE 1:**
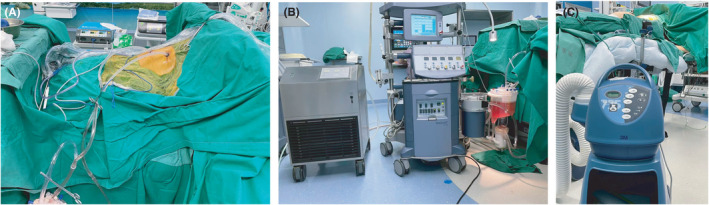
The equipment used in IPHC by VATS. (A) Chest tube. (B) The extracorporeal circulation device. (C) The hot blanket. IPHC, intrapleural perfusion with hyperthermic chemotherapy; VATS, video‐assisted thoracoscopic surgery

Patients accepted IPHC by VATS were assisted by tracheal intubation under general anesthesia, and were monitored for vital signs with electrocardiogram monitoring (ECG). Then, the patient was taken the corresponding 90 degree lateral position according to the patient's lesion position. A 3 cm incision was made along the midaxillary line at the sixth intercostal space, and an incision protector was placed. Thoracoscopy was inserted through the hole to investigate the degree of pleural adhesion and tumor involvement in the pleural cavity. After the exploration, another 1 cm operating hole was made in the fourth intercostal space along the axillary front. Thoracic adhesiolysis was performed under thoracoscope. Tumor tissue and normal pleural tissue were collected for frozen biopsy. Further immunohistochemistry and genetic testing were performed for pathological analysis after operation. After the results of frozen biopsy were reported, surgical instruments were removed. A 32F chest tube and a 28F chest tube were placed in the two operating holes as the perfusion inlet and outlet. The catheter was fixed with silk sutures, then the thoracic cavity was filled with a mixture of 80 mg/m^2^ platinum drugs and 3000–4000 ml normal saline. After then the chest tube was connected to the extracorporeal circulation device (roller pump, reservoir, and heating loop system; The details are shown in Figure 1). The mixture was gradually heated to 43°C by the heating loop system, and then circulated and closed thoracic cavity perfusion was carried out through a roller pump. The flow rate was controlled at 1000 ml/min, and a hot blanket was used to maintain the patient's pleural cavity temperature at 43°C. Each patient received about 1 h of IPHC treatment. At the end of the infusion, the fluid in the chest was progressively drained and sealed. After IPHC, thoracoscopic exploration was performed again to ensure no active bleeding or lung damage in the pleural cavity. Finally, a 32F chest tube was placed into thorax and connected to a water‐sealed drainage system. After IPHC, the patient was transferred to the intensive care unit (ICU) for further observation, and received methylprednisolone intravenous infusion and hydration treatment to prevent postoperative pulmonary edema, organ damage, and blood toxicity.

Patients who accepted NCPC were positioned by color Doppler ultrasound to determine the puncture point, and thoracentesis was performed under local anesthesia. A 10–12 cm thoracic drainage catheter fixed to the skin with local sutures was inserted, and the tube was attached to a drainage bag. Then, the pleural effusion was slowly and continuously drained (usually 2–3 days). After confirming no pleural effusion or a small amount of pleural effusion in thoracic cavity by Doppler ultrasound or x‐ray, intrathoracic perfusion treatment was performed.

### 2.4 Effectiveness and safety

In order to evaluate the curative effect of different treatments for MPE, this study evaluated the chest drainage duration, the recurrence time of pleural effusion, the control rate of pleural effusion, the toxic reactions, and the improvement for the quality of patients' life. All adverse events were reported and their severity was graded by the National Cancer Institute Universal Toxicity Criteria (version 4.0).

### 2.5 Follow‐up

Each patient had a pre‐drainage chest radiograph and computed tomography (CT). Chest radiographs were obtained twice, immediately after IPHC and 3 days later. CT would be obtained for each month after IPHC. The treatment response was identified as follows: (1) Complete response (CR) means no re‐accumulation of pleural fluid after IPHC for at least 4 weeks. (2) Partial response (PR) means recurrence of pleural effusion but below half of pre‐IPHC level for at least 4 weeks. (3) Stable disease (SD) means recurrence of pleural effusion and above half of pre‐IPHC level for at least 4 weeks but below the basic level. (4) Progressive disease (PD) means recurrence of pleural effusion and exceed the pre‐IPHC level.

### 2.6 Statistical analysis

Continuous variables are expressed as mean value ± standard deviation (SD), and independent samples *t*‐tests and nonparametric test were employed to compare the differences between different groups. Logistic regression analysis was carried out to evaluate the association between IPHC and NCPC. The chi‐squared test and the Fisher's exact test were applied to compare the distribution differences in categorical variables. End points of remission rate and PFS were measured from the date of pathological diagnosis. The Kaplan–Meier method was performed to generate the survival curves of patients who received IPHC or NCPC, and the log‐rank test was carried out to assess the differences in survival curves between two treatment groups. Cox proportional hazards method was used to further evaluate the independent factors of IPHC. All statistical analyses were processed by SPSS statistics 19.0 (IBM Corp.). A two‐tailed *p* value <0.05 was considered to be statistically significant.

## 3 RESULT

### 3.1 Clinical characteristics of patients

A total of 37 MPE patients were enrolled in this study. Twenty‐seven patients received NCPC and 10 patients received IPHC under VATS. The detailed results of clinical characteristics are shown in Table 1. There were significant differences in pathological types (*p* = 0.011), chest drainage duration (*p* = 0.005), and remission rate (*p* = 0.009) between two different treatment groups. The main pathological type in our study was lung carcinoma, while others included malignant mesothelioma, breast cancer, and esophageal cancer. The chest drainage duration of IPHC under VATS was shorter than the NCPC group (*t* = 2.969, *p* = 0.005). In IPHC group, 90% of patients were complete response (CR) and 10% of patients were partial response (PR). In NCPC group, 29.6% of patients were CR, 55.6% of patients were PR, 11.1% of patients were stable disease (SD), and 3.1% of patients were progressive disease (PD). No significant differences were observed in age, gender, smoking history, blood toxicity, and the hospital day (*p* > 0.05).

**TABLE 1:** The clinical characteristics of 37 malignant pleural effusion patients

Variables	*N* = 37 (*n*%)	I group	II group	Statistic	*p* value
Age (years)				0.240^a^	0.624
<55	9 (24.3)	6	3		
≥55	28 (75.7)	21	7		
Gender				0.509^a^	0.476
Male	22 (59.5)	17	5		
Female	15 (40.5)	10	5		
Smoking status				0.037^a^	0.847
Nonsmoker	25 (67.6)	18	7		
Smoker	12 (32.4)	9	3		
Tumor histology				7.306^a^	0.011*
Lung carcinoma	29 (78.5)	24	5		
Others	6 (16.2)	3	3		
Malignant mesothelioma	2 (5.4)	0	2		
Chest drainage duration (days)	37 (100)	9.33 ± 4.403	6.20 ± 1.989	2.969^b^	0.005*
Hospital days (days)	37 (100)	16.48 ± 15.488	13.1 ± 4.841	‐^c^	0.877
Remission rate					
Complete response	17 (45.9)	8	9	9.858^b^	0.009*
Partial response	16 (43.2)	15	1		
Stable disease	3 (8.1)	3	0		
Progressive disease	1 (2.7)	1	0		
Hematotoxicity					
None toxicity	28 (75.7)	20	8	0.139^a^	0.709
Toxicity	9 (24.3)	7	2		

Note

I group, normothermic chemoperfusion of the pleural cavity; II group, intrapleural perfusion with hyperthermic chemotherapy by VATS.

Others tumor histology include breast cancer and esophageal cancer.

^a^ Chi‐squared‐value.

^b^ *t*‐value.

^c^ Nonparametric test.

* *p* < 0.05.

### 3.2 The remission rate of MPE in different treatments

The Fisher's exact test showed that different treatments were related with the remission rate of MPE (*p* = 0.009). Binary logistic regression was modeled to estimate the relationship between the remission rate and treatments. The result showed that the remission rate of MPE in IPHC group was better than the NCPC one (OR = 0.031, 95% CI: 0.002–0.507, *p* = 0.015). Furthermore, the model also showed that the tumor histologies had no difference in IPHC and NCPC groups. The details are shown in Table 2.

**TABLE 2:** Binary regression analysis of the response rate of MPE in IPHC group and intrapleural perfusion group

Variables	*β*	Std. error	Wald	*p* value	OR	95% CI
Age (years)	−0.051	0.057	0.804	0.37	0.95	0.851–1.062
Hospital day (days)	−0.018	0.045	0.163	0.687	0.982	0.9–1.072
Gender	−0.699	1.276	0.3	0.584	0.497	0.041–6.063
Smoking status	1.113	1.244	0.8	0.371	3.043	0.266–34.851
Remission rate (trend)	−3.478	1.428	5.934	0.015*	0.031	0.002–0.507
Tumor histology (trend)	1.175	0.758	2.4	0.121	3.238	0.732–14.316

Note

I group, normothermic chemoperfusion of the pleural cavity; II group, intrapleural perfusion with hyperthermic chemotherapy by VATS.

Test of model coefficient, *p* value = 0.003.

Abbreviations: CI, confidence interval; OR, odds ratio.

* *p* < 0.05.

### 3.3 The prognosis of MPE in different treatments

**FIGURE 2:**
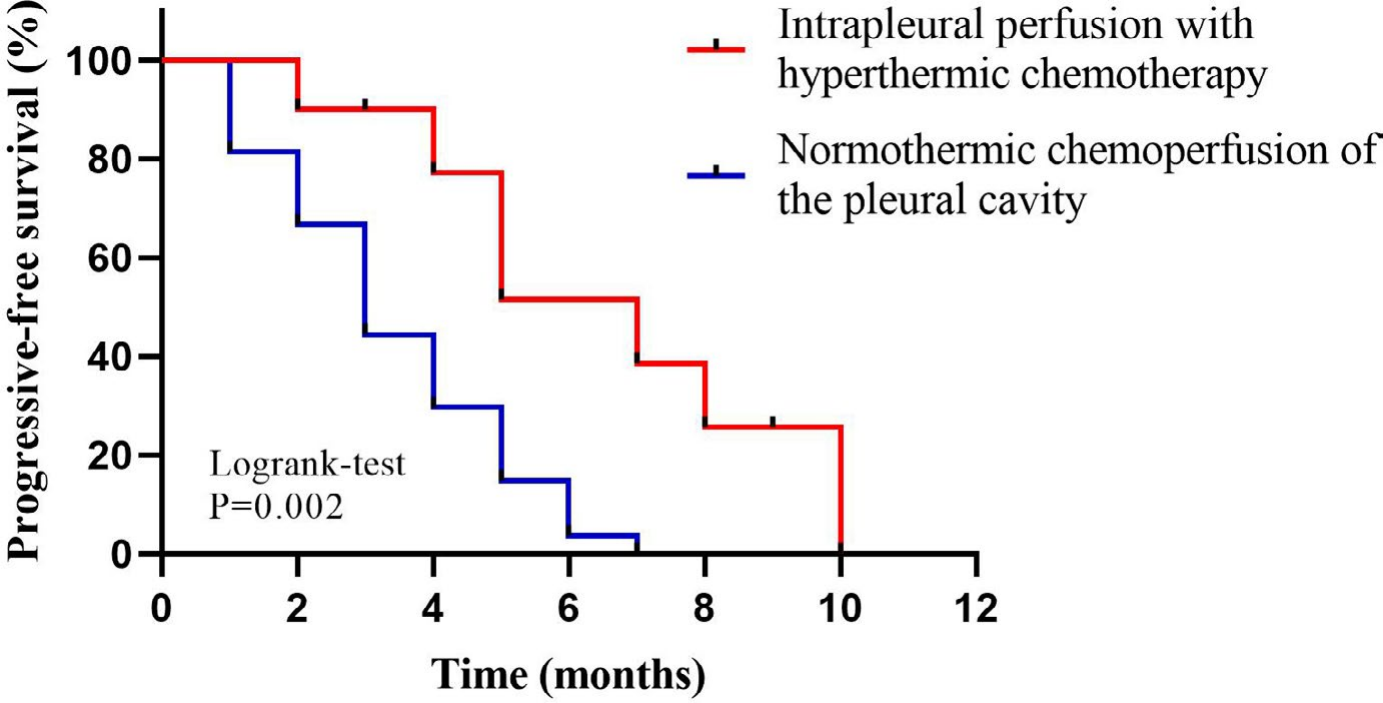
Progression‐free survival (PFS) of patients with MPE accepted NCPC or IPHC. IPHC, intrapleural perfusion with hyperthermic chemotherapy; MPE, malignant pleural effusion; NCPC, normothermic chemoperfusion of the pleural cavity

To investigate the prognosis of patients with MPE in different treatment groups, the Kaplan–Meier method was used to generate the survival curves of IPHC group and NCPC group. As shown in Figure 2, IPHC group could significantly prolong the PFS of patients with MPE compared to NCPC group (log‐rank *p* = 0.002). Univariate cox regression analysis showed that patients with MPE in the IPHC group presented significant longer PFS than that in the NCPC group (HR = 0.264, 95% CI: 0.098–0.713, *p* = 0.009). Multivariate cox regression analysis further verified this conclusion (HR = 0.268, 95% CI: 0.096–0.753, *p* = 0.012). The details are shown in Table 3.

**TABLE 3:** Association between therapy methods and PFS in MPE patients

Variables	*N* = 37	Univariate analysis			Multivariate analysis		
		HR	95% CI	*p* value	HR	95% CI	*p* value
Age (years)		1.329	0.576–3.066	0.505	1.161	0.484–2.785	0.738
<55	9						
≥55	28						
Gender		0.623	0.305–1.274	0.195	0.926	0.382–2.241	0.864
Male	22						
Female	15						
Smoking status		1.829	0.865–3.867	0.114	1.654	0.728–3.758	0.230
Nonsmoker	25						
Smoker	12						
Tumor histology		0.611	0.349–1.068	0.084	0.775	0.402–1.492	0.445
Lung carcinoma	29						
Others	6						
Malignant mesothelioma	2						
Therapy methods		0.264	0.098–0.713	0.009*	0.268	0.096–0.753	0.012*
Ⅰ group	27						
Ⅱ group	10						

Note

I group, normothermic chemoperfusion of the pleural cavity; II group, intrapleural perfusion with hyperthermic chemotherapy by VATS.

Others tumor histology include breast cancer and esophageal cancer.

Test of model coefficient, *p* value = 0.027.

Abbreviations: CI, confidence interval; HR, hazards ratio.

* *p* < 0.05.

### 3.4 Case reports

**FIGURE 3:**
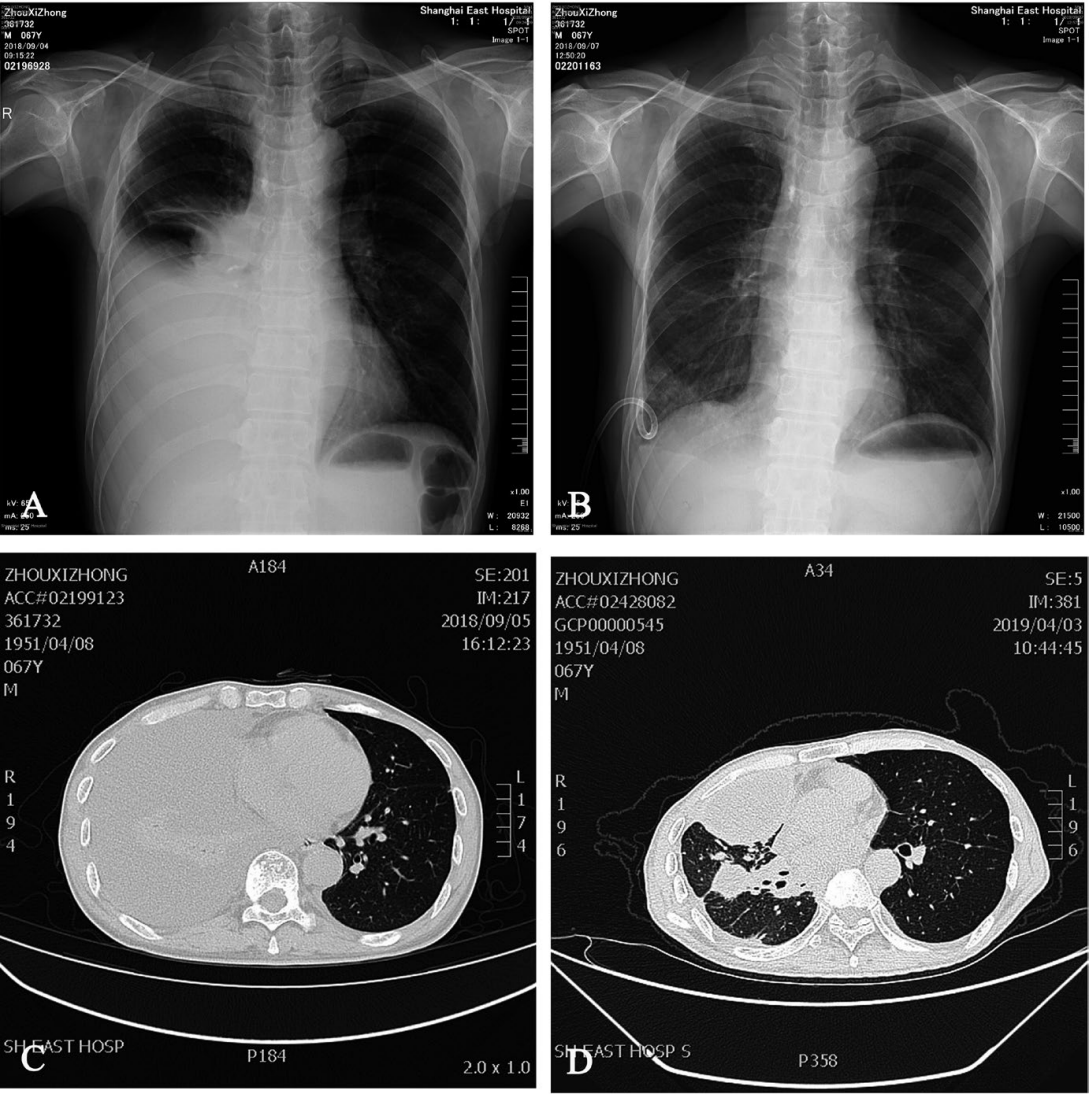
A 67‐year‐old male patient underwent IPHC by VATS and diagnosed with lung adenocarcinoma and MPE. (A) A chest radiograph showed a large pleural effusion before treatment. (B) A chest radiograph showed the pleural effusion was cured after IPHC. (C) A CT imaging showed that a large pleural effusion before treatment. (D) A CT imaging showed that a small amount of encapsulated pleural effusion after 6 months. IPHC, intrapleural perfusion with hyperthermic chemotherapy; VATS, video‐assisted thoracoscopic surgery; MPE, malignant pleural effusion

A 67‐year‐old male patient was diagnosed with MPE and underwent IPHC by VATS after a complete examination. Intraoperative stripping pleural biopsy revealed lung adenocarcinoma, pleural, and intrapulmonary metastasis. After giving cisplatin‐based IPHC under VATS, patients’ MPE was effectively controlled. On the 5th day after operation, the chest drainage tube was removed and the patient was discharged from the hospital. Re‐examination after 3 months revealed a small amount of recurrence of pleural effusion, which was an encapsulated effusion between leaves. The patient generally had no restriction on life. Re‐examination after 6 months showed no significant progress in MPE. The imaging data are included in Figure 3.

**FIGURE 4:**
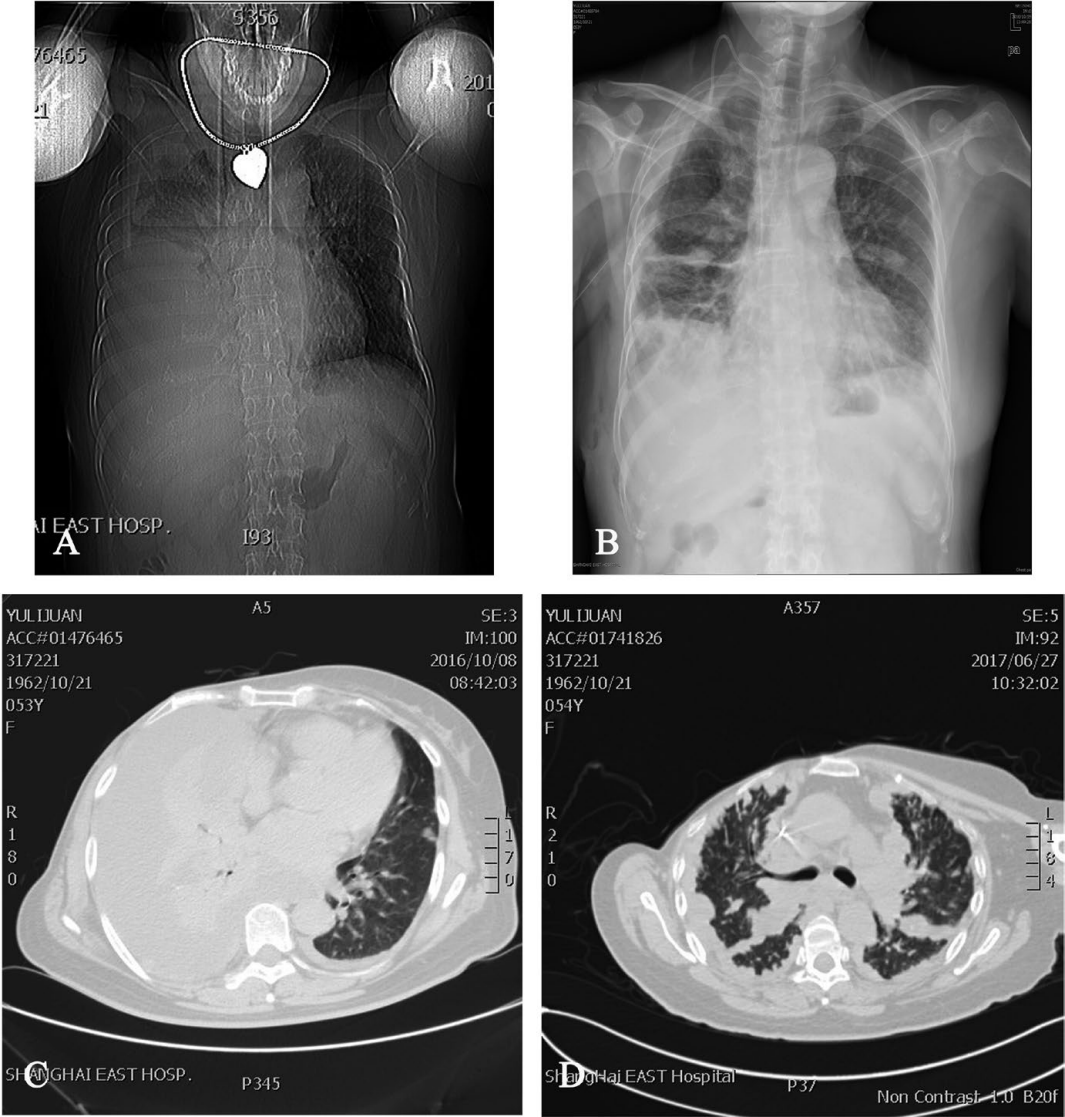
A 53‐year‐old female patient underwent IPHC by VATS and diagnosed with mucinous breast carcinoma and MPE. (A) A chest radiograph showed a large pleural effusion before treatment. (B) A chest radiograph showed the pleural effusion was cured after IPHC. (C) A CT imaging showed that a large pleural effusion before treatment. (D) A CT imaging showed that only a small pleural effusion after 9 months. IPHC, intrapleural perfusion with hyperthermic chemotherapy; VATS, video‐assisted thoracoscopic surgery; MPE, malignant pleural effusion

The other patient is a 53‐year‐old female patient diagnosed with extensive intrapulmonary metastasis of breast mucinous carcinoma with MPE. After a complete examination, IPHC by VATS was performed. The patient lived in a normal life after treatment, and the re‐examination of MPE showed no significant progress compared with the previous 1 after 6 months. The imaging data are included in Figure 4.

## 4 DISCUSSION

The treatment for MPE still has much controversy. At present, doctors commonly place a pleural drainage tube to gradually drain the pleural effusion, and infuse drug into the thorax making pleurodesis to curb MPE. However, this kind of method still has many problems. Its effective control rate of MPE and the long‐term hospitalization of patients for treatment have been criticized. Therefore, we are committed to explore a treatment that can effectively control MPE and improve the quality of patients' life. Thoracoscopy‐assisted hyperthermic perfusion chemotherapy undoubtedly meets this requirement.^1^

In this study, we found that MPE patients who received IPHC by VATS had a better prognosis than patients who received normothermic chemoperfusion of the pleural cavity (NCPC). IPHC can more effectively control the recurrence of MPE and reduce patients' hospitalization requirements and expenses, so that they can obtain a better quality of life. Furthermore, its minimally invasive makes it more easily to be used in symptomatic patients with MPE, especially those who had failed pleurodesis or did not meet the indications for pleurodesis. Admittedly, IPHC also has some complications, including infection, side effects of chemotherapy, pulmonary embolism, catheter metastasis, and so on. However, its benefits to MPE patients are more significant.

The main side effects of IPHC are nephrotoxicity and blood toxicity. Zimm S et al. reported that patients with the higher doses of cis‐platinum, more than 200 mg/m^2^, are more likely to occur renal toxicity.^3^ However, grade 2–4 renal toxicity was not observed in our study, which may be related to the low doses of platinum drugs used in this study and adequate postoperative hydration after therapy. Previous reports have shown that adequate hydration after the application of platinum drugs is helpful for the remission of renal toxicity of platinum drugs.^1^, ^3^, ^8^ In this study, we also observed that some patients had blood toxicity in both NCPC group and IPHC group, but the difference was not statistically significant. This result may due to insufficient sample size. The sample needs to be further expanded to research the blood toxicity in the future.

Compared with traditional NCPC, IPHC under VATS has the dual advantages of diagnosis and treatment. Under thoracoscopy, we can visually observe the tumor metastasis in the thoracic cavity. If the primary tumor is partly limited, palliative resection can be used to prevent the further development of the primary tumor. In addition, the pleural biopsy or pathological examination was used to identify the pathological types and the gene targets of the tumor. These clinical data will provide valuable information for subsequent treatment.^9^, ^10^, ^11^, ^12^, ^14^ At present, the minimally invasive surgery under thoracoscopy is very mature. The surgical incision is often controlled within 3 cm and the auxiliary perfusion incision is mostly 1 cm. Minimally invasive surgery with small incisions allows IPHC to be applied to patients with mild cardiopulmonary insufficiency, older age, or poor nutritional status. The improvement of surgical technique makes the trauma of patients undergo IPHC no difference with that of thoracic infusion group, and has a better curative effect. In our study, we found that the postoperative time of thoracic catheterization in patients underwent IPHC by VATS was shorter than those who accepted NCPC. This means IPHC under VATS can greatly improve the quality of patients' life during treatment. At the same time, it can help to reduce the requirement of hospitalization for patients and alleviate the economic burden.

By comparison, the traditional NCPC is more of a symptomatic treatment method for MPE, and still have a lot of controversy in the use of infusion drugs.^3^, ^4^ Platinum drugs have a good initial effect, but they are easy to cause thoracic adhesion, which is not conducive to subsequent treatment, and to increase the probability of blood toxicity at the same time.^13^ Drugs inhibiting vascular endothelial growth can quickly restrain MPE in a short period of time and have low toxicity, but the effective period is often short, and pleural effusion is prone to recurrence. Patients need frequent thoracentesis and drainage, which will reduce the quality of patient's life, and put a heavy burden on their economy and psychology. In addition, palliative pleurodesis by drug infusion is mostly a symptomatic treatment method, and many studies have shown that it cannot benefit the long‐term prognosis of patients with MPE. In Isik's study, the median survival time in the palliative pleurodesis group was only 6 months and the median survival time in the Pleuriectomy group was only 8 months. The 1‐year survival rates of the two groups were 0.6% and 0.8%. In contrast, IPHC can effectively control MPE and destroy metastatic pleural disease, which is of great significance for preventing recurrence and prolonging survival.

In our study, 90% of patients with MPE were completely controlled after receiving IPHC by VATS, and most of them could avoid recurrence of pleural effusion within 3 months, which performed better than those who received NCPC. The other scholars found that IPHC could improve the survival rate of patients with MPE. Research by Shirong Zhang and Shenglin MA et al. showed that the survival rate of 80 patients who received IPHC by VATS (median survival of 16.8 months) was significantly higher than that of patients who received pleural resection or pleural fusion. All the above data show that IPHC by VATS is better than traditional NCPC in the efficacy of controlling MPE.

We do admit that our research exists some biases and limitations due to the retrospective research method and the sample size. The MPE of patients enrolled in our study was mainly formed by lung cancer metastasis to the thoracic cavity, while we also recruited patients with MPE caused by breast tumors and other distant metastases, which may cause some heterogeneity. In addition, due to the long interval between patients enrolled in the study, the overall survival time of many patients was lost to follow‐up. Therefore, the survival rate after treatment cannot be compared. We will study the survival rate in a follow‐up study with a large sample size. In addition, we plan to add PD‐L1 biologics as a control group, and compare the efficacy and prognosis between the platinum‐based drug group and the PD‐L1 group in IPHC.^15^

In conclusion, compared with traditional NCPC, IPHC by VATS has obvious advantages of controlling MPE, shorter catheter insertion time, and longer complete remission. Moreover, the long‐term economic benefits of IPHC are also better than traditional infusion chemotherapy, which can greatly shorten the patient's hospital stay time, effectively improve the quality of patient's life, and partly benefit patient's long‐term prognosis. Therefore, we recommend IPHC as the first‐line treatment for patients with MPE who can tolerate minimally invasive surgery.

## COMPETING INTERESTS

The authors have no competing interests to declare.

## AUTHORS' CONTRIBUTIONS

Yejun Cao, Qiying Zhang, Zhengjun Chai, Jie Liu, Jinyi Wang, Zhengliang Sun, and Tian Zhao collected and checked the data of patients. Yejun Cao, Zhiyuan Huang, and Qiying Zhang carried out the data analysis. Yang Han evaluated the result of pathology and immunohistochemistry. Yejun Cao, Guohan Chen, Qinchuan Li, and Xuan Hong designed the project and wrote the manuscript.

## ETHICS APPROVAL AND CONSENT TO PARTICIPATE

The study was performed in accordance with the Declaration of Helsinki and was approved by the Ethics Committee of Shanghai East hospital.

## CONSENT FOR PUBLICATION

A written informed consent was obtained from the patients for publication of his or her clinical details and clinical images in this article.

## Data Availability

The datasets used and/or analyzed during the current study are available from the corresponding author upon reasonable request.
